# 早期肺癌特异性淋巴清扫的临床回顾研究

**DOI:** 10.3779/j.issn.1009-3419.2012.09.05

**Published:** 2012-09-20

**Authors:** 建 陈, 锋 毛, 正波 宋, 屠阳 申

**Affiliations:** 1 200030 上海，上海交通大学附属胸科医院/上海市肺部肿瘤临床医学中心 Shanghai Chest Hospital/Shanghai Lung Tumor Clinical Medical Center, Shanghai 200030, China; 2 315000 宁波，宁波市第一医院胸外科 Department of Thoracic Cancer, the First Hoipital of Ningbo, 315000 Ningbo, China; 3 310000 杭州，浙江省肿瘤医院肿瘤内科 Department of Medical Oncology, Zhejiang Tumor Hospital, Hangzhou 310000, China

**Keywords:** 肺肿瘤, 肺叶特异性淋巴结清扫, 系统性淋巴结清扫, 早期, 预后, Lung neoplasms, Lobe-specific lymph node dissection, Systematic lymph node dissection, Early stage, Prognosis

## Abstract

**背景与目的:**

本研究旨在探讨不同淋巴结清扫方式对Ⅰ期肺癌患者生存率的影响，考察影响预后的相关因素，探讨肺叶特异性淋巴结清扫的临床应用指征。

**方法:**

回顾性分析1998年-2005年上海市胸科医院病理Ⅰ期且符合完全性切除的379例肺癌患者，其中系统性淋巴结清扫组148例，肺叶特异性淋巴结清扫组150例，术后病理均为T1a-2aN0M0，比对研究两组手术相关因素并进行预后分析。

**结果:**

两组临床病理特征无统计学差异（*P* > 0.05）；两组总体3年及5年生存率无统计学差异（*P* > 0.05），但不同病理分期、病理类型和肿瘤直径之间的生存率存在明显差异（*P* < 0.01）；在手术时间、术中失血、胸管引流量、拔管时间及住院天数等方面，两组存在明显差异（*P* < 0.01）；两组术后并发症亦有统计学差异（*P* < 0.05）。

**结论:**

系统性淋巴结清扫并未增加Ⅰ期肺癌患者5年生存率；病理分期、病理类型和肿瘤直径是影响患者预后的重要因素；肺叶特异性淋巴结清扫可明显减少手术并发症并降低围手术期风险。

## Abstract (en)

**Background and objective:**

The aim of this study is to explore the effects of different modes of lymph node dissection in early-stage non-small cell lung cancer (NSCLC) on the survival rate, to study the prognostic factors, and to discuss the clinical application of lobe-specific lymph node dissection.

**Methods:**

A total of 379 patients with completely resected pathological stage Ⅰ NSCLC between the years 1998 and 2005 at Shanghai Chest Hospital were retrospectively analyzed. Among the patients, 148 were placed in the systematic lymph node dissection group and 150 in the lobe-specific lymph node dissection group. All of them were in pathological stage Ⅰ (T1a-2aN0M0). The differences between the two groups were compared.

**Results:**

No statistical difference was found between the two sets of data in terms of the clinical and pathological features of distribution (*P* > 0.05). The overall survival distribution between the two groups had no statistical difference (*P* > 0.05). The pathological stage, pathological type, and tumor size significantly differed between the two groups. The duration of surgery, blood loss, blood transfusion, drain secretion, duration of drainage, and duration of hospital stay also significantly differed between the two groups (*P* < 0.01). The complications significantly differed between the two groups as well (*P* < 0.05).

**Conclusion:**

Systematic lymph node dissection does not improve the five-year survival rates in pathological stage Ⅰ NSCLC. The pathological stage, pathological type, and tumor size critically affect the prognosis. Lobe-specific lymph node dissection can significantly reduce complications and perioperative risks.

外科手术依然是Ⅰ期、Ⅱ期和部分可切除Ⅲa期非小细胞肺癌（non-small cell lung cancer, NSCLC）的最佳治疗方式^[1-3]^，也是部分早期患者获得治愈的唯一途径。就手术方法而言，解剖性肺叶或全肺切除辅以胸内淋巴结清扫业已成为共识性的标准。但对于早期肺癌，淋巴结清扫范围和方式的选择一直存在争议^[4^-^9]^，随着小病灶肺癌发现率的逐年增加，淋巴结清扫模式的重要性愈益凸显。本研究拟回顾性分析大宗病理Ⅰ期NSCLC患者，研究不同淋巴结清扫模式对长期生存率和预后的影响，探讨早期NSCLC行肺叶特异性淋巴结清扫的可行性及其临床应用指证。

## 1 资料与方法

本研究两种淋巴结清扫方式的定义如下：①肺叶特异性淋巴结清扫术：依据肺癌原发部位制定一侧纵隔淋巴结清扫范围。右上叶或左上叶固有段肿瘤，肺门及上纵隔淋巴结未发现转移，则下纵隔淋巴结无需清扫；右中叶或左上叶舌段肿瘤，若未发现肺门及#7组淋巴结转移，则上纵隔除#R4组外，其它纵隔淋巴结可不必清扫；下叶肿瘤，未累及肺门及下纵隔淋巴结，则上纵隔淋巴结无需清扫。肺叶特异性淋巴结清扫必须切除包括#7组淋巴结在内的至少1组以上纵隔淋巴结，同时包括3组来自肺内（叶、叶间或段）和肺门淋巴结^[4-6]^。②系统性淋巴结清扫术：完整清除肿瘤所在肺叶一侧纵隔的解剖学标志之间的所有纵隔淋巴结及其周围脂肪组织。系统性淋巴结清扫必须切除6组以上淋巴结，其中3组以上须来自包括#7组在内的纵隔淋巴结。

### 1.1 临床资料

选择性收集上海市胸科医院1998年1月-2005年1月收治并行手术治疗（均为开胸肺叶或全肺切除术）的临床Ⅰ期肺癌患者共458例，术后病理Ⅰ期（2007年分期标准）且符合肺癌完全性切除病例379例（参照2005年国际肺癌研究学会肺癌完全性切除手术标准^[7]^）。对纳入病例再依据2009年分期标准^[8]^剔除：肿块直径 > 5 cm者、新辅助化疗者、术后辅助化疗者、淋巴结清扫少于3组、术后死于非肿瘤性疾病者和失访者共81例，最终纳入符合标准患者共298例，均为病理T1a-2aN0M0的NSCLC患者。其中符合系统清扫标准者148例，符合特异清扫标准者150例。以上海市胸科医院病案室存档病史为准，通过SPSS 17.0数据库采集相关研究资料。患者术后生存随访时间按月计算。随访数据来自上海市疾病预防控制中心，末次随访时间为2009年8月，中位随访时间大于5年。

### 1.2 统计学方法

采用SPSS 17.0统计软件包对数据进行处理。*t*检验分析定量资料，χ^2^检验分析总体率或构成比之间的差异，*Kaplan-Meier*乘积法和*Log-rank*检验行单因素生存分析，*Cox*回归模型行多因素生存分析。*P* < 0.05为差异有统计学意义。

## 2 结果

### 2.1 一般临床及病理特征

患者一般临床特征及构成情况如表 1所示。两组在性别、年龄、病理分期、病理类型、肿瘤直径、肿瘤分化程度、脏层胸膜侵犯及肿瘤原发部位分布等方面无统计学差异（*P* > 0.05），仅淋巴结清扫组数有明显统计学差异（*P* < 0.01）。

**1:** 所有患者一般临床特征 The clinical characteristics of all the patients

Variables	Overall^*^	Systematic-lympha denectomy^**^	Specific-lympha denectomy^**^	P^***^
Overall	298	148 (49.7%)	150 (50.3%)	
Gender				0.060
Male	182 (61.1%)	102 (56.0%)	80 (44.0%)	
Female	116 (38.9%)	46 (39.7%)	70 (60.3%)	
Age				0.630
< 60 years	107 (35.9%)	51 (47.7%)	56 (52.3%)	
≥60 years	191 (64.1%)	97 (50.8%)	94 (49.2%)	
Clinical stage				0.908
Ⅰa	132 (44.3%)	65 (49.2%）	67 (50.8%)	
Ⅰb	166 (55.7%)	83 (50.0%）	83 (50.0%)	
Histology				0.090
Ad	186 (62.4%)	76 (40.9%)	110 (59.1%)	
Sq	79 (26.6%)	50 (63.3%)	29 (36.7%)	
Ad-sq	32 (10.7%)	21 (65.6%)	11 (34.4%)	
Low-differentiated	1 (0.3%)	1 (100.0%)	0	
Tumor size				0.573
≤2 cm	56 (18.8%)	28 (50.0%)	28 (50.0%)	
> 2 cm-≤3 cm	77 (25.8%)	42 (54.5%)	35 (45.5%)	
> 3 cm-≤5 cm	165 (55.4%)	78 (47.3%)	87 (52.7%)	
Degree of differentiation				0.299
Low	42 (14.1%)	23 (54.8%)	19 (45.2%)	
Moderate	90 (30.2%)	49 (54.4%)	41 (45.6%)	
High	68 (22.8%)	26 (38.2%)	42 (61.8%)	
Middle/low	39 (13.1%)	20 (51.3%)	19 (48.7%)	
Middle/high	59 (19.8%)	30 (50.8%)	29 (49.2%)	
Visceral pleural invasion				0.131
No	139 (46.6%)	76 (54.7%)	63 (45.3%)	
Yes	159 (53.4%)	72 (45.3%)	87 (54.7%)	
Tumor location				0.441
Left upper lobe	75 (25.2%)	37 (49.3%)	38 (50.7%)	
Left lower lobe	46 (15.4%)	26 (56.5%)	20 (43.5%)	
Right upper lobe	100 (33.6%)	51 (51.0%)	49 (49.0%)	
Right middle lobe	22 (7.4%)	7 (31.8%)	15 (68.2%)	
Right lower lobe	55 (18.5%)	27 (49.1%)	28 (50.9%)	
Lymph-node-dissec- tion group number				< 0.01
< 6	152 (51.0%)	8 (5.3%)	144 (94.7%)	
≥6	146 (49.0%)	140 (95.9%)	6 (4.6%)	
^*^. Numbers in parentheses indicate percentages (accounted for the overall percentage). ^**^. Numbers in parentheses indicate percentages (compared with another group, accounted for the overall percentage). ^***^. Indicate gender, age, pathological staging, pathological types, tumor diameter, the degree of tumor differentiation, visceral pleural invasion, tumor primary site of constituent ratio showed no significant difference between two groups（*Fisher's* exact test or *X*^2^ test).				

### 2.2 总体生存状态

患者总体3年及5年生存率分别为79.9%和71.1%；其中系统清扫组分别为79.1%和73.6%；特异清扫组分别为80.7%和68.7%；两组总体3年及5年生存率比较均无统计学差异（*P* > 0.05）（图 1）。

**1:**
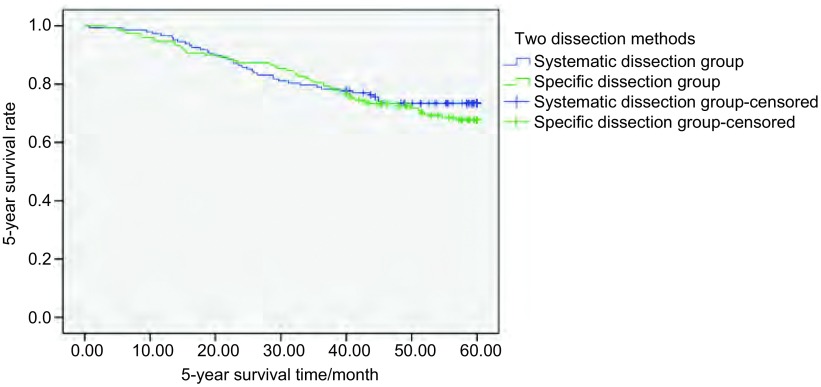
298例Ⅰ期NSCLC患者不同清扫方式5年总体生存率分析（*P*=0.414） The 5-year overall survival analysis of 298 stage Ⅰ NSCLC patients receiving different dissection methods (*P*=0.414). NSCLC: non-small cell lung cancer

### 2.3 两组不同清扫方式手术相关因素分析

两组资料围术期中手术时间、术中出血量、是否输血及输血量、术后日均胸引流量、术后拔管时间、平均住院天数比较结果见表 2所示。除输血量无统计学差异外，其它指标均存在统计学差异（*P* < 0.01）。

**2:** 两组淋巴结清扫方式围手术期相关因素分析 Analysis of the related factors during operation period in two groups of lymph node dissection

Related factors	Systematic lympha denectomy	Specific lympha denectomy	*P*
Operation time (min)	136.20±40.50	102.60±37.50	< 0.001
Intraoperative blood loss（mL）	418.00±119.00	358.00±11.60	< 0.001
Blood transfusion (mL)	300.00±109.55	300.00±141.42	0.999
Thoracic drainage (mL)	249.12±123.44	186.52±55.19	< 0.001
Extubation time (d)	3.89±0.91	3.46±0.67	< 0.001
Hospitalization time (d)	13.92±1.86	13.11±1.77	< 0.001

两组术后相关并发症如表 3所示。术后并发症共计27例，占9.06%，其中系统清扫组20例，占本组的13.5%；特异清扫组7例，占本组的4.6%。两组间手术并发症发生率有统计学差异（*P* < 0.05）。肿瘤直径≤3 cm者行系统清扫74例，其中发生肺不张、肺部感染、支气管肺泡胸膜瘘、喉返神经损伤各1例，心律失常2例，占本组的8.1%；特异清扫组60例，其中发生活动性出血、肺部感染各1例，肺不张2例，占本组的6.7%；两组手术并发症发生率无统计学差异（*P*=0.752）。肿瘤直径在 > 3 cm-≤5 cm者行系统清扫74例，其中心力衰竭、呼吸衰竭、喉返神经损伤各1例，肺不张、肺部感染各2例，心律失常4例，乳糜胸3例，占本组患者的18.9%；行特异清扫组90例，发生肺部感染1例，心律失常3例，占本组的3.3%；两组手术并发症发生率之间存在明显差异（*P*=0.001）。

**3:** 两组不同清扫方式的手术并发症 Operation complications in two groups of lymph node dissection

Operation complications	≤3 cm			> 3 cm-≤5 cm	
	Systematic-lympha denectomy (74)	Specific-lympha denectomy (60)		Systematic-lympha denectomy (74)	Specific-lympha denectomy (90)
Active bleeding	0	1		0	0
Pulmonary atelectasis	1	2		2	0
Pulmonary infection	1	1		2	1
ARDS	0	0		0	0
Arrhythmia	2	0		4	2
Heart failure	0	0		1	0
Respiratory failure	0	0		1	0
Chylothorax	0	0		3	0
Bronchoalveolar lavage, pleural fistula	1	0		0	0
Bronchial fistula	0	0		0	0
Recurrent laryngeal nerve injury	1	0		1	0
Overall	6 (8.1%)	4 (6.7%)		14 (18.9%)	3 (3.3%)

### 2.4 *Cox*回归模型多因素生存分析

将298例患者的年龄、性别、肿瘤原发部位、病理类型、肿瘤直径、肿瘤分化程度、脏层胸膜侵犯、淋巴结清扫组数、纵隔淋巴结清扫组数、淋巴结清扫方式等变量代入*Cox*回归模型进行多因素生存分析。结果提示：肿瘤直径和病理类型是影响患者生存率的重要预后因素（*P* < 0.05），患者的年龄与生存率可能相关，而纵隔淋巴结清扫方式并不是影响患者生存率的重要因素。

### 2.5 *Kaplan-Meier*单因素生存分析

总体患者中，Ⅰa期和Ⅰb期两组之间生存率存在差异（*P* < 0.01）。肿瘤直径≤2 cm、 > 2 cm-≤3 cm和 > 3 cm-≤5 cm之间生存率存在明显差异（*P* < 0.001）；腺癌、鳞癌、腺鳞癌及低分化癌之间生存率存在明显差异（*P* < 0.01）；男性、女性患者生存率亦存在统计学差异（*P* < 0.05）。而不同年龄分层、脏层胸膜侵犯、分化程度、肿瘤病灶部位等的生存率无统计学差异。

系统清扫组中，不同肿瘤直径分组间生存率存在统计学差异（*P* < 0.05）（图 2）。特异清扫组中，不同肿瘤直径分组间生存率亦存在统计学差异（*P* < 0.05），见图 3。但按不同肿瘤直径分组，采取两种不同淋巴结清扫方式的生存率无统计学差异（图 4-图 6）。

**2:**
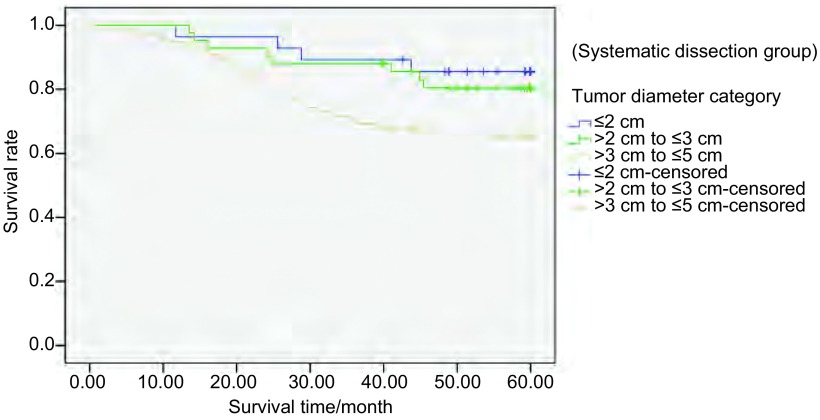
298例Ⅰ期NSCLC行系统清扫组中患者不同肿瘤直径的5年生存率（*P* < 0.05） The 5-year survival rates of 298 stage Ⅰ NSCLC patients with different tumor diameters receiving systematic dissection (*P* < 0.05)

**3:**
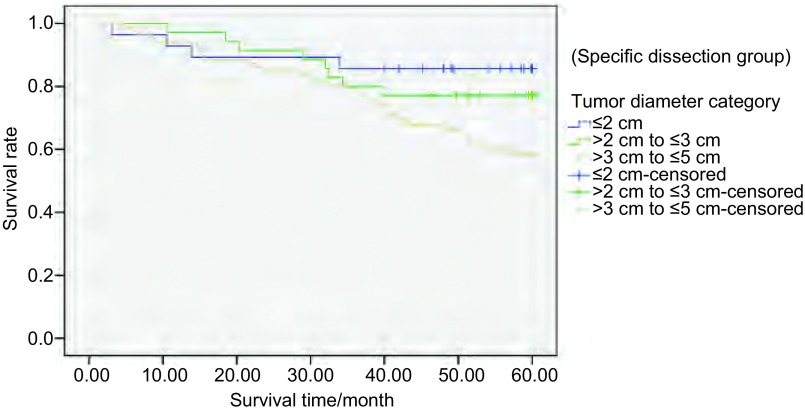
298例Ⅰ期NSCLC行特异清扫组中患者不同肿瘤直径的5年生存率（*P* < 0.05） The 5-year survival rates of 298 stage Ⅰ NSCLC patients with different tumor diameters receiving specific dissection (*P* < 0.05)

**4:**
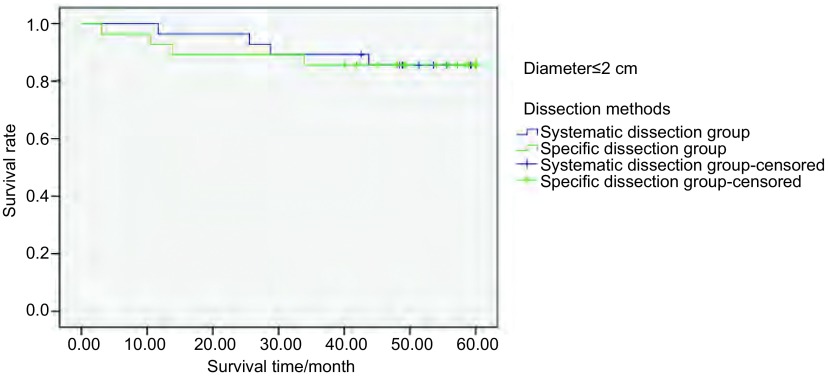
298例Ⅰ期NSCLC肿瘤直径≤2 cm患者行不同清扫方式的5年生存率分析（*P*=0.950） The 5-year survival rate analysis of 298 stage Ⅰ NSCLC patients (tumor diameter≤2 cm) treated with different dissection methods (*P*=0.950)

**5:**
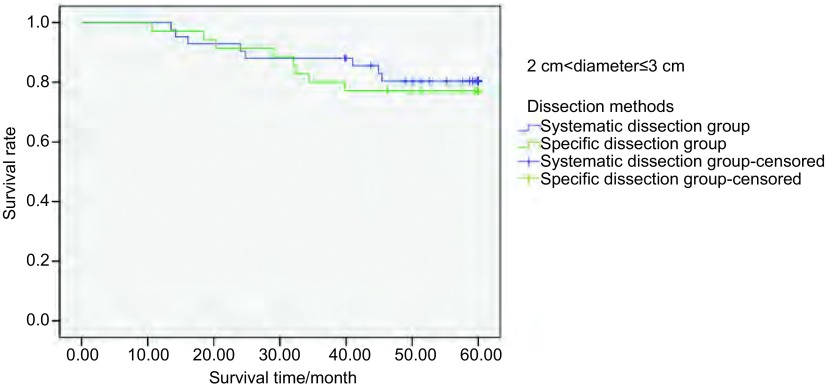
298例Ⅰ期NSCL肿瘤直径 > 2 cm-≤3 cm患者行不同清扫方式的5年生存率分析（*P*=0.698） The 5-year survival rate analysis of 298 stage Ⅰ NSCLC patients (2 cm < tumor diameter≤2 cm) treated with different dissection methods (*P*=0.698)

**6:**
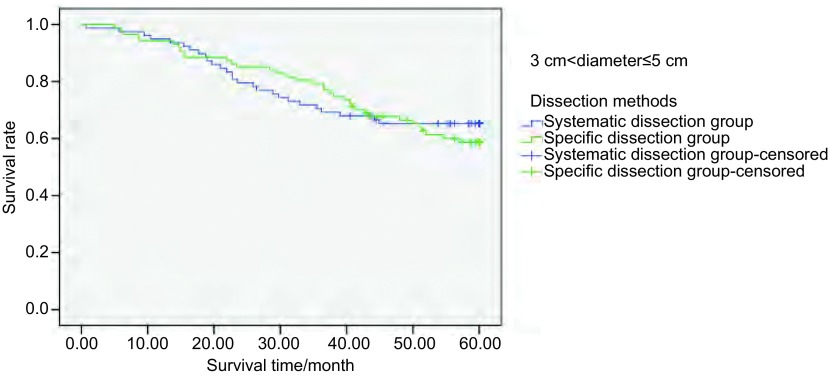
298例Ⅰ期NSCLC肿瘤直径 > 3 cm-≤5 cm患者行不同清扫方式的5年生存率分析（*P*=0.642） The 5-year survival rate analysis of 298 stage Ⅰ NSCLC patients (3 cm < tumor diameter≤5 cm) treated with different dissection methods (*P*=0.642)

## 3 讨论

本研究中，两组在性别、年龄、病理分期、病理类型、肿瘤直径、肿瘤分化程度、脏层胸膜侵犯及肿瘤原发部位等方面的分布无统计学差异，提示两组临床病例资料具有可比性。总体病理类型之间存在明显差异（*P* < 0.01），其中腺癌占62.42%，鳞癌26.51%，腺鳞癌10.74%，符合早期肺癌多为周围型病灶，且以腺癌居多的病理特征。术中两组淋巴结清扫组数符合两清扫方式各自对纵隔淋巴结清扫组数的最低要求。

本研究总体中，病理分期为Ⅰa期和Ⅰb期者生存率存在明显差异（*P* < 0.01）；肿瘤直径≤2 cm、 > 2 cm-≤3 cm和 > 3 cm-≤5 cm者的生存率存在明显差异（*P* < 0.001），与国际肺癌T分期结果相似，进一步提示肿瘤直径是影响患者预后的重要独立因素。

淋巴结清扫方式是否有利于患者生存率的改善，目前仍然存在争议。随着肺癌早期诊断技术和理念的进展更新，临床Ⅰ期病例逐年增多，事实证明多数早期患者鲜见非区域淋巴结转移，促使胸外科医师反思系统性淋巴结清扫对此类患者的必要性。对于Ⅰ期肺癌淋巴结的清扫方式，目前争议的焦点在于系统性淋巴结清扫是否有利于患者生存率的改善及分期。Okada等^[4]^将临床Ⅰ期肺癌患者分为两组，一组377例行肺叶特异性淋巴结清扫，另一组358例行系统性淋巴结清扫，结果显示，在无病生存率和总生存率方面，两种淋巴结清扫方式无明显差异。多因素分析提示肺叶特异性淋巴结清扫对无病生存率无明显影响，两组术后相关病死率、肿瘤远处转移率和局部复发率无明显差异。作者由此认为，对临床Ⅰ期肺癌行肺叶特异性巴结清扫可以达到与系统性淋巴结清扫相同的临床疗效，且前者更符合外科的微创理念。相反，吴一龙等^[9]^报道了526例肺癌手术患者进行的前瞻性随机试验研究，其中268例行系统性淋巴结清扫，另264例行采样，结果显示前者的5年生存率较后者有明显提高，提示行系统性淋巴结切除对患者预后的益处。但其研究病例不仅限于临床Ⅰ期，还包括较高比例的Ⅱ期、Ⅲ期病例，且其对照组为系统性淋巴结采样。

本研究中，两组间患者总体3年及5年生存率均无统计学差异（*P* > 0.05），提示系统性淋巴结清扫并未给患者带来更多的生存获益，多因素分析提示清扫方式并不是影响生存率的重要因素（*P* > 0.05）。将肿瘤直径作为分层依据，结果表明不同直径肿瘤两种清扫方式的5年生存率无统计学差异（*P* > 0.05），进一步提示两种清扫方式对患者生存率的影响并无明显差异。有研究对临床Ⅰa期和病理T1患者的淋巴结清扫方式进行了对比分析，结果显示：两组的总生存率和无病生存率无明显差异，对于早期N0患者，系统性淋巴结清扫未能使患者有更多生存获益，而系统性淋巴结采样可以达到与系统性淋巴结清扫相同的长期生存率^[10]^。同时，该研究还发现，肿瘤直径≤2 cm者和肿瘤直径 > 2 cm-≤3 cm者的预后存在差异：肿瘤直径≤2 cm者，两组的5年总生存率和无病生存率相似；肿瘤直径 > 2 cm-≤3 cm者，系统性淋巴结清扫组的5年总生存率和年无病生存率分别为81.6%和77.9%，而系统性淋巴结采样组分别为55.8%和52.5%。作者因此建议，对临床Ⅰ期肺癌，可由术中病理测量肿瘤直径，再采用不同的淋巴结清扫方式，即细化T1a和T1b区别对待的策略。

一般认为，系统性淋巴结清扫有利于发现淋巴结微转移灶，从而使术后病理分期更准确，然而术式对肺癌分期的确定性作用并未被广泛接受。曾有两项研究报道系统性淋巴结清扫有利于提高肿瘤分期的准确性，但鉴于一项研究中无对照组^[11]^，而另一研究对照组的淋巴结清扫数量极其有限^[12]^，故结论可容置疑。相反，一些研究者发现，系统性淋巴结清扫对提高N分期的精确性并无明显优势，如Izbicki等^[13]^及Keller等^[14]^发现，无论系统性淋巴结清扫较系统性淋巴结采样所清除的淋巴结数量增加多少，术后两组病理发现的N1或N2病例数百分比相近。

本研究提示两组手术相关并发症发生率存在明显差异，系统清扫可明显增加手术并发症的发生率，且乳糜胸及喉返神经损伤等较严重并发症的发生率明显提高，而肺叶特异性淋巴结清扫可明显降低手术并发症的发生率。国内亦有研究^[9, 10]^报道系统性淋巴结采样能够减少手术并发症，降低围手术期风险。Sugi等^[15]^对115例直径 < 2 cm的周围型肺癌分别行系统性淋巴结清扫和系统性淋巴结采样[*n* (59:56)]，此项随机性研究证实与系统性清扫相关的病死率明显高于系统性采样（23.8% *vs* 3.4%），作者认为对于肿瘤直径 < 2 cm的周围型肺癌不需要行系统性淋巴结清扫。而Keller等^[14]^和Izbicki等^[16]^则报道，与系统性采样相比，系统性清扫较并未增加手术并发症率，围术期风险亦无增高。本研究对围手术期相关因素进一步的分析发现：两种清扫方式在手术时间、术中失血、胸管引流量、拔管时间及住院天数方面存在明显差异（*P* < 0.001），提示系统清扫较特异清扫的围手术期风险增高。Wright等^[17]^对3项行系统性淋巴结清扫和淋巴结采样的前瞻性研究进行了*meta*分析，结果提示行系统性淋巴结清扫的患者术后死亡风险有所减小，HR=0.78（95%CI: 0.65-0.93, *P*＝0.005），然而术中淋巴结切除是否有生存获益仍然存在争议，其确切性尚不清晰。在第90届美国胸外科年会上，Gail等^[18]^公布了一项北美多中心合作的大规模前瞻性随机研究结果：系统性纵隔淋巴结清扫对于经术中采样阴性的早期肺癌患者并没有明显的生存率改善，同时也未减少肿瘤的局部和远处复发率，进一步给早期肺癌淋巴结清扫方式的选择提供了参考。

## 4 结论

基于本研究数据，对T1a-2aN0M0的早期非小细胞肺癌患者，系统性淋巴结清扫并未有更多的长期生存获益，而肺叶特异性淋巴结清扫可明显减少手术并发症的发生率且可降低围手术期风险，可视为更高效减创的手术方式。本文仅为临床回顾性研究，因此我们仍期待更具说服力的大规模前瞻性随机对照研究的证据，对愈益增多的早期病例转而采用肺叶特异性淋巴结清扫，也或是一种理性的回归，此非惟科学，尤着眼于患者的福祉，值得关注和探究。
